# Membranous aplasia cutis congenita in trisomy 18

**DOI:** 10.1186/s13052-020-00885-6

**Published:** 2020-08-27

**Authors:** Francisco Cammarata-Scalisi, Andrea Diociaiuti, Blanca de Guerrero, Colin Eric Willoughby, Michele Callea

**Affiliations:** 1 Pediatrics Service, Regional Hospital of Antofagasta, Antofagasta, Chile; 2 Dermatology Unit, Bambino Gesù Children’s Hospital, IRCCS, Rome, Italy; 3 Foundation Child Development Center, Mérida, Venezuela; 4 Biomedical Sciences Research Institute, Ulster University, Northern Ireland, UK; 5 Unit of Dentistry, Bambino Gesù Children Hospital and Research Institute, Rome, Italy

**Keywords:** Aplasia cutis congenita, Membranous aplasia cutis congenita, Trisomy 18, Defective closure of the neural tube

## Abstract

**Background:**

Aplasia cutis congenita (ACC) is a rare congenital condition characterized by the absence of skin layers and sometimes other underlying structures, in a localized or widespread area. The exact etiopathogenesis is not yet completely understood. Membranous ACC (MACC) also described as bullous or cystic ACC is a clinical subtype of ACC, covered with a membranous or glistening surface, and appears as a flat scar. There are less than 20 cases reported in the literature. It has been proposed an abortive form of a defective closure of the neural tube. On the other hand, the trisomy 18 is a chromosomal abnormality characterized by a broad clinical spectrum and the presence of defective closure of the neural tube.

**Case presentation:**

We report on an 18-months-old Venezuelan boy, who presented on the parietal scalp a distinctive localized MACC appearing as an oval lesion covered with a membranous surface, characterized by the absence of hairs and the presence of a sharp hair collar. The karyotype in peripheral blood was 47,XY,+ 18.

**Conclusions:**

This is the second case report of ACC in trisomy 18 and reinforces the interpretation of a non-fortuitous association as well as of a defective closure of the neural tube as pathogenetic mechanism. The case highlights the importance of examining for dermatological alterations such as ACC in cases of chromosomopathy.

## Background

Aplasia cutis congenita (ACC) is a rare congenital condition characterized by the absence of skin layers and sometimes other underlying structures, in a localized or widespread area [1–3]. The exact etiology of ACC is not completely understood. Several factors have been proposed as possible causes, e.g., vascular disruption, thrombotic events, trauma, amniotic defects, chromosomal abnormalities or gene mutations, ectodermal dysplasia, defective closure of the neural tube, and teratogenic events such as intrauterine infections, or drugs and medications during pregnancy [4–6].

The most common lesion location of ACC is the scalp in 70% [7, 8]; however, any skin site can be affected [7]. The estimated incidence of ACC is approximately 0.5–3 in 10,000 newborns [2, 5], regardless of gender or ethnicity [5]. The clinical appearance of ACC is heterogeneous and it can be associated with various systemic congenital anomalies. However, in most cases, it is just a sporadic defect and the cause is unknown [2, 3].

Membranous ACC (MACC) also described as bullous or cystic ACC is a clinical subtype of ACC, covered with a membranous or glistening surface [3, 8], and appears as a flat scar. There are less than 20 cases reported in the literature [3]. A review of 17 MACC cases identified that all lesions were located on the skull with the majority on the vertex or parietal scalp, ranging in number from 1 to 7 lesions, and frequently associated with bone defects 6/17 [9]. We present the second case of ACC associated with trisomy 18 to emphasize that the association might not be fortuitous and that the pathogenesis may reflect a defective closure of the neural tube.

## Case report

An 18-months-old Venezuelan boy, first case of ACC in the family, born from nonconsanguineous healthy parents, (44-year-old mother at the time of conception). The child was delivered from the sixth mother’s pregnancy. The prenatal course was complicated with a threatened abortion evidenced as transvaginal bleeding due to a uterine myoma. The infant was born at 41 weeks by caesarean section and the birth weight was 1830 g, (SD − 3.7), height 40 cm (SD − 8.1), and head circumference 31 cm (percentile < 3). Apgar test was 6 and 8 points at the first and fifth minute respectively.

**Fig. 1:**
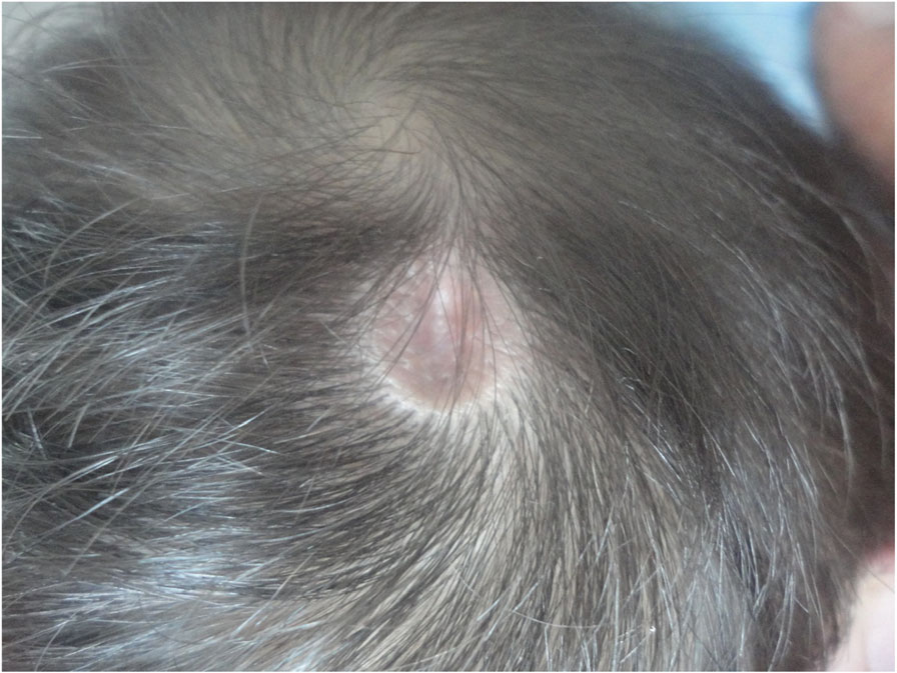
MACC oval lesion covered with a membranous surface

**Fig. 2:**
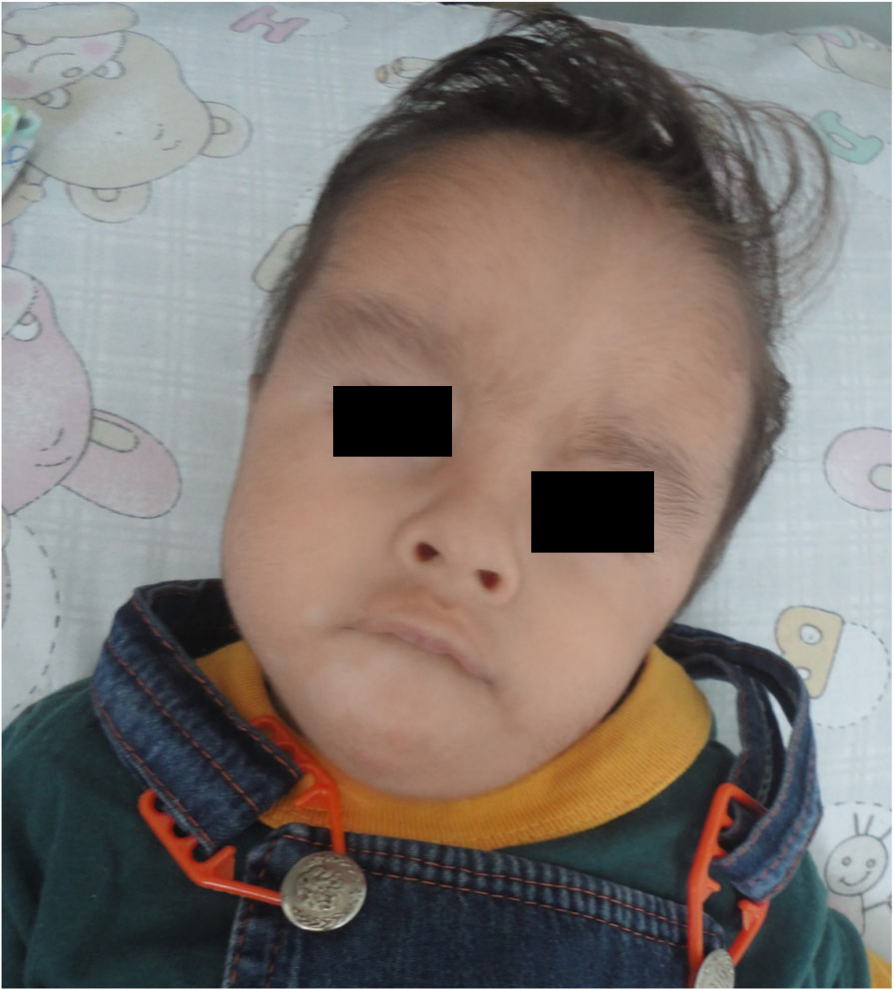
Prominence at the level of the metopic suture, wide frontal region with hypertrichosis and anteverted nostrils

At 8 months of age the infant was 4570 g in weight (SD − 5.3), size 56 cm (SD − 0.4), head circumference 40.5 cm (percentile < 3). The normotensive anterior fontanelle was of 2 × 2 cm with high implantation of the hair and an oval lesion covered with a membranous surface of 1.3 × 1.1 cm on the parietal scalp characterized by absence of hair and presence of a sharp hair collar (Fig. 1). Transfontanellar sonography showed no abnormalities. Facial dysmorphism, prominence at the level of the metopic suture, wide frontal region with hypertrichosis, convergent bilateral strabismus, depressed nasal bridge and anteverted nostrils (Fig. 2), hypoplasia and proximal insertion of thumb with equine varus right foot and valgus left foot were present. Moreover, the infant presented heart sounds with systolic murmur grade III/VI, and right cryptorchidism.

Echocardiography showed ostium secundum type interatrial communication, interventricular communication, and persistent arterial duct with hemodynamic repercussion. The karyotype in peripheral blood was 47,XY,+ 18, metaphases studied 30.

### Discussion and conclusions

Drolet et al. [10]. have proposed that MACC is an abortive form of a defective closure of the neural tube and that the hair collar sign should be regarded as a relatively specific marker for this defect [11]. Associated findings were hydrocephaly, meningeal arteriovenous fistula, epilepsy, spasticity, primary optic nerve atrophy, corneal lipodermoid changes, cornea scleralization, cleft palate, hemangioma and nevus flammeus [8]. More recently two features have been reported as more specific or pathognomonic “hair bulbs arranged radially along hair-bearing margins” [12], and the “golf club set” on trichoscopy [3].

The diagnosis of MACC is essentially clinical, and a biopsy is usually not needed. MACC can be visualized on prenatal ultrasonography as a smooth cystic lesion without flow. Ultrasonography of the lesion and transfontanellar ultrasonography can rule out skull defects or cerebral alterations. Conservative therapy is the option of choice [3, 8].

Trisomy 18 is a chromosomal abnormality most of the times incompatible with life and a limited survival. The clinical spectrum is broad, more than 130 different abnormalities having been described in these patients and the presence of defective closure of the neural tube among patients with trisomy 18 is not uncommon [13]. In a previous study on a series of mosaic trisomy 18, we did not find ACC in five cases studied [14].

Genetics plays a pathogenetic role in ACC as indicated by its occurrence in other chromosomopathies (Table 1, Supplementary), as well as with mutations such as the ribosomal GTPase *BMS1* gene [15]. In addition, familial cases of ACC have been reported with an autosomal dominant transmission [16].

ACC and trisomy 18 have been described first in an Indian newborn [17], who died 5 days after birth. The occurrence of an additional case would support that the association is not fortuitous and that the pathogenesis may reflect a defective closure of the neural tube. Our child was 18 months old at the time of detection of the chromosomal abnormality. The long survival indicates a mild-moderate form with most malformations correctable.

The case highlights the importance of examining the entire integument to search for dermatological alterations such as ACC and making a complete assessment of multisystemic lesions, especially in cases of a mild form of chromosomopathy and a long survival.

## Supplementary information

**Additional file 1 Supplementary file 1 Table 1.** Chromosomal alterations described in the ACC, divided into numerical and structural.

## Data Availability

The datasets used and/or analyzed during the current study are available from the corresponding author on reasonable request.

## Abbreviations

ACC: Aplasia cutis congenita
MACC: Membranous aplasia cutis congenita
