# Supplementary material for: Membranous aplasia cutis congenita in trisomy 18
Source: Ital J Pediatr. 2020 Aug 27;46:120. doi: 10.1186/s13052-020-00885-6 (PMC7450555; doi:10.1186/s13052-020-00885-6)
Supplement: Supplementary file 1 — Additional file 1 Supplementary file 1 Table 1. Chromosomal alterations described in the ACC, divided into numerical and structural. [file 13052_2020_885_MOESM1_ESM.docx]

Table 1. Chromosomal alterations described in the ACC, divided into numerical and structural.

| Author | Sex | Ethnicity | Localization | Chromosomal alteration |
| --- | --- | --- | --- | --- |
|  |  |  |  | **Numeric** |
| Puvabanditsin et al [1] | M | American | Scalp | Trisomy 13 |
| Mıhçı et al [2] | M | Turkish | Scalp | Trisomy 13 |
| Meza et al [3] | M | Peruvian | Scalp | Trisomy 13 |
| Cuadra et al [4] | F | Chilean | Scalp | Trisomy 13 |
| Maillet-Declerck et al [5] | NS | French | Scalp | Trisomy 13 |
| Kosnik & Sayers [6] | F | American | Scalp | Trisomy D |
| Guanti et al [7] | F | Italian | Scalp | Trisomy D |
| Mishra [8] | F | Indian | Scalp | Trisomy 18 |
| Brosnan et al [9] | F | American | Scalp | 46,XY gonadal dysgenesis |
| Moro et al [10] | M | Chinese | Scalp | Klinefelter syndrome  Incontinentia pigmenti |
|  |  |  |  | **Structural** |
| Guthrie et al [11] | F | American | Scalp | Deletion 4p |
| Hirschhorn et al [12] | M | American | Scalp | Deletion 4p |
| Schierz et al [13] | NS | Italian | Scalp | Duplication 10p15.1 |
| Zakowski et al [14] | F | American | Neck and axilla | Tetrasomy 12p |
| [O'Riordan](https://pubmed.ncbi.nlm.nih.gov/?sort=date&size=50&term=O'Riordan+AM&cauthor_id=27826649) et al [15] | M | Irish | Scalp | Deletion 15qter |
| Malan et al [16] | M | French | Scalp | Deletion 19q13.11 |
| Schierz et al [13] | NS | Italian | Scalp | Duplication 19q12 |
| Zvulunov et al [17] | F | Israeli | Face and neck | Deletion Xp 22 |
| Lee et al [18] | F | Korean | Scalp | 46,XX, t(6;18)(q23.2;q11.2) |
| Khan et al [19] | M | Asian | Scalp, face, chest, back, elbows, hands,ankles and feet, | Trisomic 1qter and monosomic 12qter |

M: male, F: female, NS: not specified

**References**

1. Puvabanditsin S, February M, Garrow E, Bruno C, Mehta R. Our experience with a severe case of aplasia cutis congenita with a large skull defect. Int J Dermatol. 2016;55(10):1151‐3.

2. Mihçi E, Erişir S, Taçoy S, Lüleci G, Alpsoy E, Oygür N. Aplasia cutis congenita: three cases with three different underlying etiologies. Turk J Pediatr. 2009;51(5):510‐4.

3. Meza Méndez B, Barboza Martínez J, Beltrán Grados G, Santos Rurya R, Victorero Montani E, Elezcano Concha S, et al. Aplasia cutis congénita tipo 9 asociada a trisomía 13 ó síndrome de Patau. Dermatol Peru. 2003;13(2):136-8.

4. Cuadra M, Brain M, Werner K, Weitz C, Garrido C, Gac K. Aplasia cutis congénita: reporte de cuatro casos. Rev Ped Elec. [en línea] 2011;8(2):7.

5. Maillet-Declerck M, Vinchon M, Guerreschi P, Pasquesoone L, Dhellemmes P, Duquennoy-Martinot V, et al. Aplasia cutis congenita: review of 29 cases and proposal of a therapeutic strategy. Eur J Pediatr Surg. 2013;23(2):89‐93.

6. Kosnik EJ, Sayers MP. Congenital scalp defects: aplasia cutis congenita. J Neurosurg. 1975;42(1):32‐36.

7. Guanti G, Del Sordo F, Petrinelli P, Battaglia E. A Case of Trisomy D1 — Syndrome of Patau, Caryologia, 1970;23(4):489-99.

8. Mishra S, Agarwalla SK, Praharaj BR, Potpalle DR. Edward syndrome with aplasia cutis congenita: A rare case report. IJNMR. 2014;2(3):7-9.

9. Brosnan PG, Lewandowski RC, Toguri AG, Payer AF, Meyer WJ. A new familial syndrome of 46,XY gonadal dysgenesis with anomalies of ectodermal and mesodermal structures. J Pediatr. 1980;97(4):586‐90.

10. Moro R, Fabiano A, Calzavara-Pinton P, Cardinale J, Palumbo G, Giliani S, et al. Incontinentia Pigmenti Associated with Aplasia Cutis Congenita in a Newborn Male with Klinefelter Syndrome: Is the Severity of Neurological Involvement Linked to Skin Manifestations?. Dermatol Ther (Heidelb). 2020;10(1):213‐20.

11. Guthrie RD, Aase JM, Asper AC, Smith DW. The 4p- syndrome. A clinically recognizable chromosomal deletion syndrome. Am J Dis Child. 1971;122(5):421‐5.

12. Hirschhorn K, Cooper HL, Firschein IL. Deletion of short arms of chromosome 4-5 in a child with defects of midline fusion. Humangenetik. 1965;1(5):479‐82.

13. Schierz IAM, Giuffrè M, Del Vecchio A, Vincenzo Antona V, Corsello G, Piro E. Recognizable neonatal clinical features of aplasia cutis congenita. Ital J Pediatr. 2020;46:25.

14. Zakowski MF, Wright Y, Ricci A Jr. Pericardial agenesis and focal aplasia cutis in tetrasomy 12p (Pallister-Killian syndrome). Am J Med Genet. 1992;42(3):323‐5.

15. O'Riordan AM, McGrath N, Sharif F, Murphy NP, Franklin O, Lynch SA et al. Expanding the clinical spectrum of chromosome 15q26 terminal deletions associated with IGF-1 resistance. Eur J Pediatr. 2017;176(1):137‐42.

16. Malan V, Raoul OF, Firth HV, Royer G, Turleau C, Bernheim A, et al. 19q13.11 deletion syndrome: a novel clinically recognizable genetic condition identified by array-CGH. J Med Genet. 2009;46(9):635-40.

17. Zvulunov A, Kachko L, Manor E, Shinwell E, Carmi R. Reticulolinear aplasia cutis congenita of the face and neck: a distinctive cutaneous manifestation in several syndromes linked to Xp22. Br J Dermatol. 1998;138(6):1046‐52.

18. Lee EH, Park TS, Choi YS, Cho EH. Familial aplasia cutis congenita associated with mega-cisterna magna. Pediatr Int. 2016;58(10):1054‐6.

19. Khan JY, Moss C, Roper HP. Aplasia cutis congenita with chromosome 12q abnormality. Arch Dis Child Fetal Neonatal Ed. 1995;72(3):F205‐6.
